# Correlates of Trachoma Recrudescence: Results from 51 District-Level Trachoma Surveillance Surveys in Amhara, Ethiopia

**DOI:** 10.3390/tropicalmed9120298

**Published:** 2024-12-05

**Authors:** Eshetu Sata, Nicholas A. Presley, Phong Le, Andrew W. Nute, Zebene Ayele, Ayalew Shiferaw, Demelash Gessese, Ambahun Chernet, Berhanu Melak, Tania A. Gonzalez, Kimberly A. Jensen, Adisu Abebe Dawed, Taye Zeru, Zerihun Tadesse, Elizabeth Kelly Callahan, Scott D. Nash

**Affiliations:** 1 Trachoma Control Program, The Carter Center, Addis Ababa 1169, Ethiopia; eshetu.sata@cartercenter.org (E.S.); zebene.ayele@cartercenter.org (Z.A.); ayalew.shiferaw@cartercenter.org (A.S.); demelash.gessese@cartercenter.org (D.G.); ambahun.chernet@cartercenter.org (A.C.); berhanu.melak@cartercenter.org (B.M.); zerihun.tadesse@cartercenter.org (Z.T.); 2 Trachoma Control Program, The Carter Center, Atlanta, GA 30307, USA; nicholas.presley@cartercenter.org (N.A.P.); pahongle@gmail.com (P.L.); awnute@gmail.com (A.W.N.); tania.gonzalez@cartercenter.org (T.A.G.); kim.jensen@cartercenter.org (K.A.J.); kelly.callahan@cartercenter.org (E.K.C.); 3 Health Research Development Directorate, Amhara Public Health Institute, Bahir Dar 641, Ethiopia; adisua0@gmail.com (A.A.D.); zerutaye@gmail.com (T.Z.)

**Keywords:** trachoma, *Chlamydia trachomatis*, survey, recrudescence, Ethiopia

## Abstract

Trachoma recrudescence is a serious concern for trachoma control programs. Programs define recrudescence as the return of trachomatous inflammation-follicular (TF) prevalence above elimination threshold (≥5%) on district-level trachoma surveillance surveys (TSSs). This study aimed to determine potential correlates of trachoma recrudescence within a historically highly endemic region. Between 2015 and 2021, population-based TSSs were conducted in 51 districts of Amhara, Ethiopia. District estimates were calculated accounting for multistage design; logistic regression was used to estimate the association of key correlates with recrudescence at the district level. Among the 51 districts, 17 (33%) were recrudescent. Correlates of recrudescence included indicators of historic trachoma burden, such as higher trachomatous inflammation-intense (TI) prevalence (odds ratio [OR]: 2.6, CI: 1.4–5.3) and higher *Chlamydia trachomatis* (*Ct*) infection prevalence (OR: 2.9, CI: 1.1–9.9) at the first recorded impact survey. The increased prevalence of children with clean faces (OR: 0.4, CI: 0.21–1.0) and the increased prevalence of travel time to a water source ≤ 30 min (OR: 0.5, CI: 0.2–1.1) at the TSS were associated with a protective effect from recrudescence. Data on historical trachoma burden as well as current water and sanitation conditions may help programs predict where recrudescence is more likely to occur and thus help programs sustain elimination as a public health problem.

## 1. Introduction

Trachoma is caused by the bacterium *Chlamydia trachomatis* (*Ct*) [1]. It is the leading infectious cause of blindness, particularly in low- and middle-income countries with poor sanitation and limited access to healthcare. The World Health Organization (WHO) recommends the SAFE strategy for eliminating trachoma as a public health problem, consisting of surgery for correcting trachomatous trichiasis (TT), antibiotics to clear *Ct* infection, and facial cleanliness and environmental improvement to reduce *Ct* transmission [1]. The Trachoma Control Program in Amhara, Ethiopia, has implemented the SAFE strategy regionwide since 2010. Considerable progress has been made, as many districts have reached the WHO elimination threshold (<5%) for trachomatous inflammation-follicular (TF) prevalence among children ages 1 to 9 years during population-based trachoma impact surveys (TISs) [2]. In agreement with WHO guidelines, when districts reach the elimination threshold for TF, a trachoma surveillance survey (TSS) is conducted at least two years later to determine whether the prevalence of TF has remained below 5% [3].

Recrudescence is a major threat to the global elimination of trachoma. Recrudescent TF has recently been defined in a WHO-convened meeting as “an enumeration unit with at least one TSS at which the TF among children ages 1 to 9 years is ≥5%” [4]. Unfortunately, recrudescent TF has been observed in many previously endemic districts across the world, with the problem particularly acute in Ethiopia, where, as of 2021, 56% of TSSs have resulted in TF ≥ 5% [4,5]. When a district is designated as having recrudescent TF, mass drug administration (MDA) with antibiotics is typically restarted, and additional TISs and TSSs are required. Therefore, recrudescent TF results in increased costs to programs and considerable delays in countries’ ability to reach the WHO threshold for elimination as a public health problem. While the percentage of TSSs that was classified as recrudescent through 2019 was lower in Amhara (24%) than the national average (56%), it still constitutes a considerable challenge to the program’s goal of trachoma elimination as a public health problem by 2030 [2].

The aim of this study was to better understand the correlates of recrudescence (≥5% TF at the TSS) at the district level. Indicators such as the historical burden of trachoma, historical levels of ocular *Ct* infection, programmatic MDA indicators, and current water and sanitation (WASH) indicators were selected based on their biological mechanisms that may contribute to recrudescence as well as their use as key indicators for Trachoma Control Programs worldwide.

## 2. Materials and Methods

### 2.1. Ethical Clearance

The Ethical Review Committee of the Amhara Regional Health Bureau and the Institutional Review Board of Emory University reviewed and approved the protocols for all surveys described in the manuscript. Because illiteracy was high throughout Amhara, verbal informed consent and assent was allowed by the review boards. Survey protocols and methodology were reviewed by Tropical Data staff (2017 through 2021) (https://www.tropicaldata.org/, [Accessed on 1 October 2024]).

### 2.2. Survey Design

For TISs and TSSs conducted between 2010 and 2017, the methodology has been described extensively [6,7,8,9]. During this period, surveys were population based, used a multistage cluster sampling approach, and were performed by certified trachoma graders. These surveys were conducted to measure progress following 3 to 8 years of the SAFE strategy.

From 2017 to 2021, Tropical Data became the standard for trachoma surveys globally, specifically throughout Ethiopia, including the Amhara region. Methodology for Tropical Data has been described in previous publications and is similar to the methods used in earlier years in Amhara [10]. In brief, a multistage design was implemented, of which the first stage consisted of sampling villages that were chosen through a probability proportional to estimated size approach. The second stage consisted of segmenting villages and randomly choosing segments of approximately 30 households. All individuals ≥ 1 year old were enumerated, and all present and consenting individuals were examined for clinical signs of trachoma. Due to the demographics of Amhara, 30 clusters of 30 households were sampled in almost all districts.

### 2.3. Training Graders/Recorders

From 2010 to 2017, the same grader training methods were used for all surveys conducted. These methods have been described in previous publications and required all graders to be certified to grade TF prior to the survey [6,7,9]. From 2017 to 2021, the grader training did not differ drastically from those provided in the past. Graders were required to pass both a slide-based inter-grader assessment (IGA) and a field-based IGA consisting of identifying trachoma signs present in 50 children. A passing score for a grader trainee consisted of a kappa of at least 0.7 for TF when compared to a master grader [10]. Graders were also trained in collecting conjunctival swabs. Three-day training specifically given to data recorders was provided before every survey, which included both classroom and field exercises. This training consisted of lessons on how to use survey software, the consent process, and segmentation of villages. Trainees were required to pass an examination prior to data collection.

### 2.4. Data Collection/Grading

From 2010 to 2017, data were entered on electronic tablets using Swift Insights software (version 1.1), while from 2017 to 2021, data were entered on Android phones using the Tropical Data application [6,10]. A household-level questionnaire was given that collected indicators related to WASH. Indicators for WASH were defined in alignment with WHO/United Nations International Children’s Emergency Fund (UNICEF) Joint Monitoring Program for improved and unimproved water sources and sanitation facilities [11]. An improved water source was defined as one of the following: protected spring, hand pump/tube, well/borehole, public piped water/tap/standpipe, private piped into yard/dwelling, or rainwater collection. An improved latrine was defined as one of the following: flush or pour-flush to piped sewer system, septic tank pit latrines, ventilated improved pit latrines, or pit latrines with slab or composting toilets. Access to a water source within 30 min included the retrieval of water from any source, improved or otherwise [6,10]. The presence of a clean face was defined as the prevalence of children ages 1 to 9 years with neither ocular nor nasal discharge, as determined by the graders.

Graders used ×2.5 magnification loupes and ample lighting to examine eligible individuals for signs of trachoma using the WHO simplified grading system [12]. Identified cases of active trachoma (TF and/or trachomatous inflammation-intense [TI]) were offered treatment with 1% tetracycline eye ointment to be used twice daily for 6 weeks according to current WHO guidelines and identified cases of TT were referred to the nearest health post for surgical services. MDA coverage was measured using the Amhara Trachoma Control Program’s programmatic records.

### 2.5. Swabbing/Infection Testing

Conjunctival swabbing as part of these TISs and TSSs in Amhara has been described previously [7,9]. Briefly, consenting examined children ages 1 to 5 years in surveyed households were swabbed to test for the presence of ocular *Ct*. Gloved graders swabbed the upper tarsal conjunctiva firmly 3 times with a polyester-tipped swab (Fisher Scientific, Waltham, MA, USA), rotating 120 degrees with each swab. After swabbing, the grader placed the swab into a 2.0 mL tube, which was then kept in a cooler bag with ice packs. Samples were then stored in the laboratory in −20 °C freezers until testing.

All laboratory testing was performed at the Trachoma Molecular Laboratory, Amhara Public Health Institute (APHI), in Bahir Dar, Ethiopia. Laboratory technicians, who were masked to both the district of origin and the trachoma status of the children, processed the samples. Conjunctival swabs from each district were randomized, and five samples were combined to form each pool. The pools were processed with the RealTime polymerase chain reaction (PCR) assay on the Abbott m2000 system to estimate the district-level prevalence of infection [13]. The RealTime assay targets 2 conserved targets on the *Ct* plasmid.

### 2.6. Statistical Analysis

Data manipulation and analysis were conducted in R using the *tidyverse* package library [14,15]. Graphics were prepared in R using *ggplot2* [16], and maps were illustrated using ArcGIS Pro 3.2.0 [17]. Recrudescent TSS was defined as TF prevalence ≥ 5% during TSS. For the 7 districts with only 1 TIS prior to receiving a TSS, prevalence for TF, TI, and *Ct* from the first TIS were applied to both first and most recent TIS.

In the descriptive analysis, mean and standard deviation were calculated for the previously described indicators, which were further disaggregated by TSS outcome. In the bivariable analysis, logistic regressions were run to calculate the odds ratios (ORs) for recrudescence. Trachoma prevalence indicators of TF, TI, and *Ct* were derived into ordered categorical variables, corresponding to the first and most recent TIS. For trachoma prevalence indicators collected during the first TIS, WHO-defined categories were used for TF (<5%, 5–9.9%, 10–29.9%, ≥30%) [18]. Arbitrary categories based on data distribution were used for TI (<2%, 2–4%, 4–6%, ≥6%). For values corresponding to the last TIS, arbitrary categories were established for TF (<2.5%, 2.5–3.5%, 3.5–4.5%, ≥4.5%) and TI (<0.2%, 0.2–0.44%, 0.44–0.7%, ≥0.7%) because of the low prevalence. As no thresholds for *Ct* have been developed, *Ct* was derived into categories for both first and last TIS, indicating whether a district contained 0% infection, infection that was below the median, or infection that was above the median. Current WASH variables (from the TSS) were transformed into ordered categorical variables based on tertiles, indicating whether a district falls into the top tertile, middle tertile, or bottom tertile of the overall distribution.

After the initial logistic regressions were run, a multivariable logistic regression was run with all indicators associated with recrudescence, assuming a cutoff *p*-value of 0.1 and no pre-existing concerns of collinearity. In both the bivariable and multivariable analysis, all variables, including those initially defined in tertiles or as categorical variables, were treated as continuous and should be interpreted as trend tests across the categories. This approach was primarily driven by the relatively small number of districts available for this analysis. As *Ct* and TI prevalence have continually been demonstrated to be highly correlated at the district level in Amhara, 2 multivariable models were evaluated, the first including *Ct* infection from the first TIS as a more direct reflection of ongoing *Ct* transmission and the second substituting TI in for *Ct*, as it is a more commonly collected outcome for trachoma programs [7,8]. In both models TF was retained because of its status as the most common outcome used by programs.

## 3. Results

**Figure 1:**
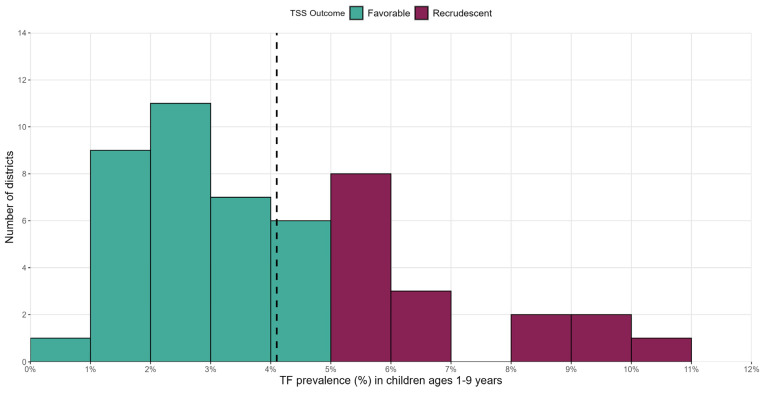
Histogram of 51 trachoma surveillance surveys (TSSs) by district-level TF prevalence among children ages 1 to 9 years, Amhara, Ethiopia, between 2015 and 2021. Favorable TSSs are those that returned a trachomatous inflammation-follicular (TF) prevalence of <5% among children ages 1 to 9 years, while recrudescent corresponds to TSSs with TF prevalence ≥ 5%. The dashed vertical line corresponds to the overall mean across all 51 districts (4.1%).

**Figure 2:**
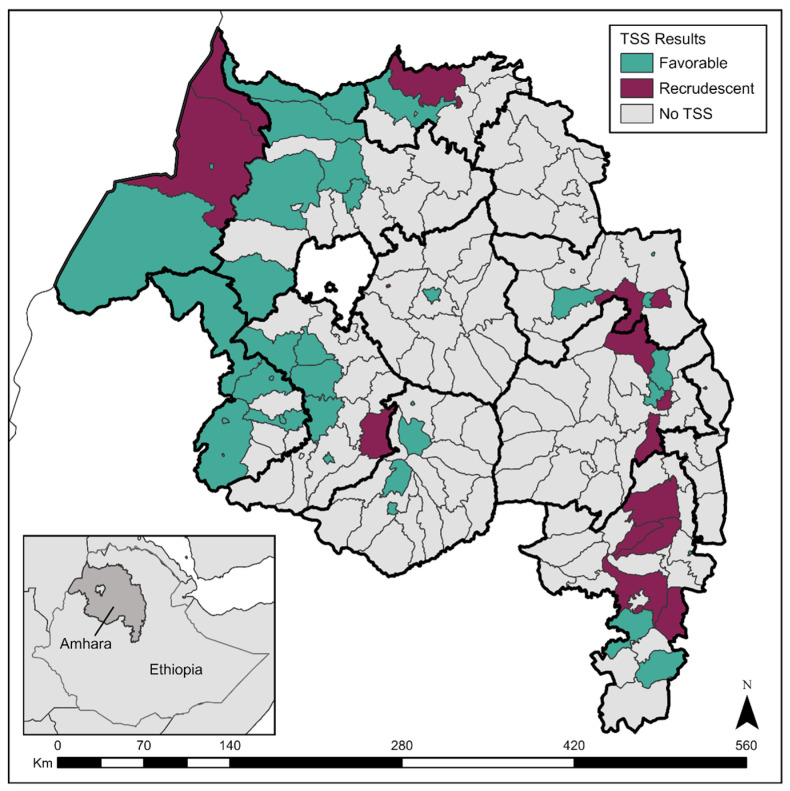
Districts that received a trachoma surveillance survey (TSS) in Amhara, Ethiopia between 2015 and 2021. Favorable TSSs are those that returned a trachomatous inflammation-follicular (TF) prevalence of <5% among children ages 1 to 9 years, while recrudescent corresponds to TSSs with TF prevalence ≥ 5%. Map created in ArcGIS Pro 2.2.6 (ESRI, Redlands, CA, USA) using a shapefile sourced from the GADM database.

Between 2015 and 2021, 51 districts had undergone a TSS in Amhara. These surveys encompassed 1446 clusters, 47,356 households, and 180,306 children ages 1 to 9 years examined for the clinical signs of trachoma. During these TSSs, the mean TF prevalence among children ages 1 to 9 years was 4.1%, and the mean TI prevalence was 0.4% (Table 1). Although all districts had TF and TI prevalence data during their TSS, *Ct* infection data was only collected during a TSS in 8/51 (15.7%), of which a mean prevalence of 0.3% was found. Across all 51 TSSs, 17 (33%) were recrudescent (TF ≥ 5%) with estimates ranging from 5.0% to 11.2% TF (Figure 1). The median year of TSSs for both recrudescent and favorable districts was 2019 (Supplemental Figure S1). Recrudescent districts were concentrated in the southeast of the region (Figure 2). In contrast, amongst the districts that had received a TSS in the northwest, only three were recrudescent.

**Figure 3:**
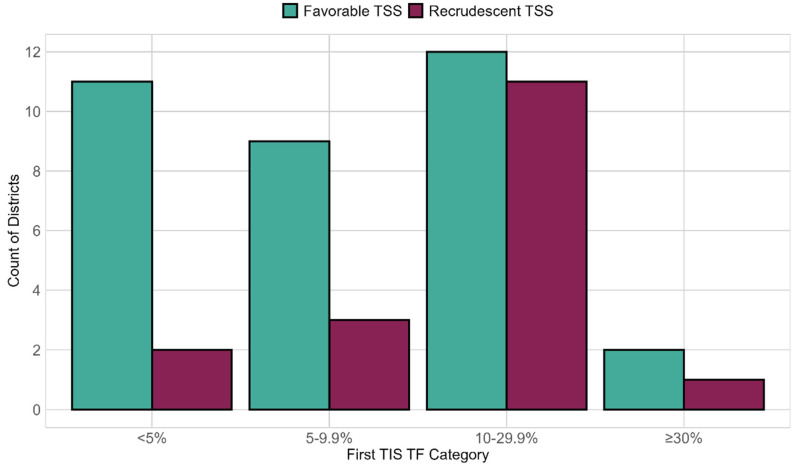
Outcome of 51 trachoma surveillance surveys (TSSs) by district-level trachomatous inflammation-follicular (TF) prevalence category during the first trachoma impact survey (TIS), Amhara, Ethiopia, between 2015 and 2021. Favorable TSSs are those that returned a trachomatous inflammation-follicular (TF) prevalence of <5% among children ages 1 to 9 years, while recrudescent corresponds to TSSs with TF prevalence ≥ 5%.

Districts with a favorable TSS outcome tended to have lower trachoma prevalence during their first TIS than recrudescent districts. At the first TIS, districts with favorable TSS results had a mean TF prevalence of 12.5%, while recrudescent districts had a mean TF prevalence of 15.0%. Those districts that, during their first TIS, had recorded a TF prevalence in the higher WHO categories (≥10–29.9% and ≥30%) were recrudescent at the TSS (12/26, 46%) more often than those in the lower TF categories, TF ≥ 5–9.9% (3/12, 25%) and <5% (2/13, 15%) (Figure 3). Districts with a favorable TSS had a mean prevalence of 2.6% for TI and 0.2% for *Ct* at the first TIS, while recrudescent districts had a mean prevalence of 3.8% for TI and 0.9% for *Ct*.

For current WASH indicators, districts with favorable TSS results had a mean prevalence of 58.9% for latrine access (improved latrines 16.1%), 66.0% for an improved water source, and 62.7% for access to a water source within 30 min. This was higher than recrudescent districts, where the mean district prevalence was 54.1% for latrine access (improved latrines 12.6%), 63.8% for an improved water source, and 55.6% for access to a water source within 30 min. The prevalence of clean face among children ages 1 to 9 years was 75.2% in districts with a favorable TSS and 70.0% in recrudescent districts. No districts returned recrudescent TSS results when the clean face prevalence was greater than 77% (Supplemental Figure S2). Differences between non-recrudescent and recrudescent districts were observed for the percentage of total MDAs that had less than 80% coverage (14.1% compared to 4.8%), number of TISs performed (3.1 compared to 3.7), and number of previous rounds of MDA (7.9 rounds compared to 9.7 rounds).

The bivariable analysis found that the burden of trachoma at the first TIS, such as TI prevalence (OR: 2.6, 95% confidence interval [CI]: 1.4–5.3; per categorical increase) and *Ct* prevalence (OR: 2.9, CI: 1.1–9.9, per categorical increase), were important correlates of recrudescence (Table 2). A higher TF prevalence at the first TIS was suggestive of recrudescence (OR: 1.9, CI: 1.0–4.1; per WHO treatment categorical increase). There was quite strong evidence that programmatic indicators, including the total number of surveys performed (OR: 2.3, CI: 1.1–5.3; per additional survey) and total number of previous rounds of MDA (OR: 1.8, CI: 1.2–2.9; per additional MDA), were associated with increased odds of recrudescence. There was suggestive evidence that the prevalence of a clean face at the TSS has a protective effect from recrudescence (OR: 0.5, CI: 0.2–1.0, per tertile increase), as did access to a water source within 30 min (OR: 0.5, CI: 0.2–1.1, per tertile increase).

In the first multivariable model, after controlling for other variables, access to a water source within 30 min at the TSS (OR: 0.2, CI: <0.1–0.5, per categorical increase) and *Ct* prevalence at the first TIS (OR: 4.9, CI: 1.3–27.8; per categorical increase) remained important correlates of recrudescence, while TF at the first TIS and a clean face at the TSS were suggestive (Table 3). In the second model, which replaced TI for *Ct* at the first TIS, access to a water source and TI were found to be important correlates of recrudescence.

## 4. Discussion

Recrudescent trachoma is a serious concern for trachoma control programs, as it necessitates restarting MDA interventions and multiple subsequent additional surveys to monitor progress. Understanding the correlates of recrudescence, and thus having a better idea of which districts may be at risk of recrudescence, is essential for developing strategies to sustain trachoma elimination. In Amhara, historically higher levels of *Ct* infection and TI prevalence were strongly associated with recrudescence, with suggestive evidence of a correlation between historical levels of TF and recrudescence. As more districts with historically high trachoma burdens reach the WHO elimination threshold of TF < 5% and plan their own TSSs, program managers should anticipate an increase in recrudescence when compared to historically lower endemic districts, underscoring the importance of maintaining surveillance in these high-risk areas.

The history of the Trachoma Control Program in Amhara highlights considerable progress while also drawing attention to the complexities of reaching and sustaining the elimination of trachoma as a public health problem in regions with historically high trachoma prevalence. The Amhara Trachoma Control Program implemented its first TSS in 2015 across five districts, of which none were recrudescent despite being surrounded by higher endemic districts [9]. As the Amhara Program continued SAFE implementation and districts that had a higher historical trachoma prevalence began to reach the elimination threshold, the proportion of TSSs that were recrudescent began to rise [2]. Of the 51 districts that had received a TSS by 2021, 17 (33%) were recrudescent. Considering the high baseline prevalence of trachoma throughout Amhara, the considerable *Ct* infection still remaining in the region, and that 108/166 (65.1%) of districts are still endemic (TF ≥ 5%) [2,19,20], it is likely that as more districts reach the elimination threshold, the proportion of districts with recrudescent TSS results in Amhara will increase. As the recrudescent rate in Ethiopia overall has been even higher than that observed in Amhara (56% vs. 33%), recrudescence will continue to be a considerable problem in Ethiopia going forward [5].

After adjusting for TF at the first TIS, a clean face, and travel time to water, our multivariable model found the prevalence of *Ct* infection at the first TIS to be strongly associated with recrudescence. This highlights the role that *Ct* infection data can play as a complementary indicator for trachoma surveillance, especially in locations where MDA has been implemented [21]. Ocular swabbing to measure *Ct* infection has been incorporated into trachoma surveys in Amhara since 2011 and continues to be a key indicator the Trachoma Control Program in Amhara uses to monitor progress [7,19]. The benefits of having *Ct* infection data available to programs have become more widely recognized, particularly in districts where recrudescence has been observed or is suspected [4]. In areas where *Ct* infection monitoring may not be feasible due to the associated costs, may not be feasible due to programmatic reasons, and/or was not collected during the early stages of MDA implementation, TI may be a suitable alternative. This was observed both in its performance in our bivariable and second multivariable models as well as the previously demonstrated correlation with *Ct* infection [7,8]. Statistically significant associations were not observed between *Ct* infection prevalence at the last TIS and recrudescence, likely because of the low *Ct* prevalence when TF is <5%. The importance of low levels of infection once the elimination threshold has been reached has yet to be determined [9].

While our study identified the importance of some current WASH variables on favorable TSS results, the results were less definitive than those variables directly related to trachoma. Many household-level WASH indicators, such as access to a latrine or an improved water source, did not demonstrate strong associations with TSS outcomes. In fact, after controlling for other variables, the only household-level WASH variable that showed a strong association was access to a water source within 30 min (OR: 0.2, CI: <0.01–0.5). Water access has been observed to be an important correlate of trachoma globally and has been previously associated with trachoma hotspots in Amhara [22,23]. One explanation for why other household-level WASH variables were not significantly correlated could be related to the need for more precise indicators to measure these variables. For example, it has been theorized that directly observed household water quality and water use for hygiene could be a more informative indicator for health surveys [22,24]. The prevalence of a clean face, which has been associated with trachoma hotspots in the region previously, had suggestive evidence of an association with recrudescence in this study [22]. Facial hygiene likely plays a critical role in reducing trachoma transmission, and interventions to improve facial cleanliness are a key part of the SAFE strategy. The Trachoma Control Program has implemented numerous interventions focused on facial cleanliness over many years, including community health education, water point construction, and a region-wide school trachoma program [6,20,25]. A sustained commitment to WASH improvement is likely the best way to avoid trachoma recrudescence over the long term.

Our study had a few key limitations. First, our study’s scope was limited to 51 districts in Amhara. Because of the limited size of our data set, it was not possible to perform more complex statistical testing, such as multilevel effect modification. Similarly, while analyzing categorical variables as continuous within logistic models assumes a linear relationship, this method was ultimately chosen to optimize the use of limited events in our data (recrudescence, n = 17) as well as improve model stability. As more TSSs are conducted in Ethiopia and the sample size increases, these insights can be refined, and future iterations may re-evaluate these assumptions and explore more complex relationships. Finally, given most trachoma control programs use standardized TIS/TSS methods and variables, future incorporation of results from TSSs from other countries may lead to deeper insights into trachoma recrudescence.

This study relied on the newly accepted definition of recrudescence, which is based on the WHO TF ≥ 5% prevalence threshold. While programmatically relevant, this threshold may be epidemiologically arbitrary and should not be confused with biological recrudescence (return of sustained *Ct* transmission). Districts just above 5% are not likely to be epidemiologically different from those just below. Sampling variation, statistical randomness, or a misdiagnosis of TF by the survey teams could incorrectly place borderline districts on either side of the elimination threshold. To address these issues, districts with a TF prevalence between 5–9.9% at a TSS in Ethiopia are now being considered for a “wait and watch” approach, whereby a follow-up survey with complementary indicators is conducted, approximately a year after the TSS, to determine whether MDA truly needs to be restarted [2,4]. As more trachoma programs begin to collect data on *Ct* infection and serology, future guidelines could consider more nuanced criteria in defining recrudescence, balancing the need for prompt action against the risk of excessive MDA and survey implementation.

## 5. Conclusions

This study provided critical insights into the correlates of trachoma recrudescence in the Amhara region of Ethiopia. Districts with historically higher trachoma burdens, including higher levels of *Ct* infection and currently lower levels of water availability, should be targeted for more intensive interventions and should be more carefully monitored for recrudescence over time. By enhancing our understanding of recrudescence, the Trachoma Control Program in Amhara and the global program can develop more effective strategies to not only reach but sustain the elimination of trachoma as a public health problem.

## Figures and Tables

**Table 1:** Descriptive analysis of 51 trachoma surveillance surveys (TSSs) in Amhara, Ethiopia, between 2015 and 2021.

	Favorable TSS ^1^	Recrudescent TSS ^2^	Overall
Variable	Mean (SD)	Mean (SD)	Mean (SD)
General WASH Indicators at TSS (%)			
Children with clean faces	75.2 (12.9)	70.0 (4.6)	73.4 (11.0)
Access to a latrine	58.9 (26.0)	54.1 (28.6)	57.8 (26.3)
Access to an improved latrine	16.1 (18.7)	12.6 (17.5)	14.9 (18.2)
Access to an improved water source	66.0 (21.4)	63.8 (19.4)	65.3 (20.6)
Access to a water source within 30 min	62.7 (22.3)	55.6 (20.1)	60.3 (21.6)
First TIS Prevalence			
TF	12.5 (12.8)	15.0 (10.2)	13.3 (12.0)
TI	2.6 (6.1)	3.8 (2.3)	3.0 (5.2)
*Ct*	0.2 (0.7)	0.9 (1.5)	0.4 (1.1)
Most Recent TIS Prevalence			
TF	3.5 (2.7)	3.0 (1.2)	3.3 (2.3)
TI	0.8 (0.9)	0.4 (0.3)	0.6 (0.8)
*Ct*	0.2 (0.6)	0.5 (0.9)	0.3 (0.7)
TSS Prevalence			
TF	2.7 (1.1)	7.0 (2.2)	4.1 (2.5)
TI	0.4 (0.5)	0.6 (0.6)	0.4 (0.5)
MDA Coverage			
% Of Total MDA < 80% Coverage	14.1 (24.5)	4.5 (6.7)	11.0 (20.8)
Number of Surveys Performed	3.1 (0.8)	3.7 (0.9)	3.3 (0.9)
Number of Previous Rounds of MDA	7.9 (2.1)	9.7 (1.3)	8.5 (2.0)
Number of High TF Prevalence Clusters During Most Recent TIS			
Greater than 5%	1.9 (2.4)	3.1 (2.1)	2.3 (2.4)
Greater than 10%	0.5 (1.1)	0.7 (0.8)	0.6 (1.0)
Number of High TF Prevalence Clusters During TSS			
Greater than 5%	0.9 (1.0)	3.9 (2.3)	1.9 (2.1)
Greater than 10%	0.1 (0.4)	1.5 (1.3)	0.6 (1.0)

*Ct*—*Chlamydia trachomatis* infection. MDA—Mass drug administration. %—percent. SD—Standard deviation. TF—trachomatous inflammation-follicular. TI—trachomatous-inflammation intense. TIS—Trachoma impact survey. TSS—Trachoma surveillance survey. WASH—Water, sanitation, and hygiene. TSS surveys with *Ct* data excluded from descriptive analysis due to small sample size (n = 8). ^1^ Favorable TSS are those which returned a TF prevalence of <5% among children ages 1 to 9 years. ^2^ Recrudescent TSS corresponds to TSS with TF prevalence ≥ 5% in children ages 1 to 9 years.

**Table 2:** Summary of bivariable model for predictors of recrudescent trachoma surveillance survey (TSS) results based on 51 TSSs from Amhara, Ethiopia, between 2015 and 2021.

	Bivariate Analysis	
Variable	OR (95% CI)	*p*-Value ^1^
General WASH Variables (Tertial)		
Children with clean faces	0.5 (0.2, 1.0)	0.07
Access to a latrine	0.8 (0.3, 2.0)	0.64
Access to an improved latrine	0.9 (0.4, 1.8)	0.72
Access to an improved water source	1.1 (0.6, 2.4)	0.72
Access to a water source within 30 min	0.5 (0.2, 1.1)	0.08
First TIS Prevalence (Categorical)		
TF	1.9 (1.0, 4.1)	0.08
TI	2.6 (1.4, 5.3)	0.01
*Ct*	2.9 (1.1, 9.9)	0.05
Most Recent TIS Prevalence (Categorical)		
TF	0.7 (0.4, 1.3)	0.29
TI	0.6 (0.3, 1.0)	0.07
*Ct*	2.1 (0.7, 7.4)	0.19
MDA Coverage		
% of Total MDA < 80% Coverage ^2^	0.7 (0.3, 1.0)	0.16
Number of Surveys Performed ^3^	2.3 (1.1, 5.3)	0.03
Number of Previous Rounds of MDA ^3^	1.8 (1.2, 2.9)	<0.01

CI—Confidence intervals. *Ct*—*Chlamydia trachomatis* infection. MDA—Mass drug administration. OR—Odds ratio. %—percent. TF—trachomatous inflammation-follicular. TI—trachomatous-inflammation intense. TIS—Trachoma impact survey. WASH—Water, sanitation, and hygiene. All variables included in the model, including those originally defined in tertiles or as categorical variables, were treated as continuous. ^1^ Wald’s Test. ^2^ Scaled by a factor of 10 to facilitate interpretation of the model coefficients. ^3^ Although *p*-values are below 0.1 threshold in bivariable analysis, they were not included in multivariable model due to TF’s role as a confounder on “number of surveys performed” and “number of previous rounds of MDA”.

**Table 3:** Summary of multivariable models for predictors of recrudescent trachoma surveillance survey (TSS) results based on 51 TSSs from Amhara, Ethiopia, between 2015 and 2021.

	Multivariable Analysis			
	Model #1: with *Ct*		Model #2: with TI	
Variable	OR (95% CI)	*p*-Value ^1^	OR (95% CI)	*p*-Value ^1^
General WASH Variables (Tertial)				
Children with clean faces	0.3 (0.1, 0.9)	0.04	0.4 (0.1, 1.1)	0.07
Access to a water source within 30 min	0.2 (<0.1, 0.5)	<0.01	0.3 (0.1, 0.7)	<0.01
First TIS Prevalence (Categorical)				
TF	3.6 (1.0, 18.9)	0.05	1.7 (0.6, 5.7)	0.32
TI			2.5 (1.1, 6.8)	0.03
*Ct*	4.9 (1.3, 27.8)	0.02		

CI—Confidence intervals. *Ct*—*Chlamydia trachomatis* infection. MDA—Mass drug administration. OR—Odds ratio. %—percent. TF—trachomatous inflammation-follicular. TI—trachomatous-inflammation intense. TIS—Trachoma impact survey. WASH—Water, sanitation, and hygiene. All variables included in the model, including those originally defined in tertiles or as categorical variables, were treated as continuous. ^1^ Likelihood Ratio Test (LRT).

## Data Availability

Data underlying the findings of this study are owned by Amhara Regional Health Bureau. De-identified data are available upon request. Requests to access data should be directed to the corresponding author.

## Supplementary Materials

The following supporting information can be downloaded at https://www.mdpi.com/article/10.3390/tropicalmed9120298/s1, Figure S1: Histogram of 51 Trachoma Surveillance Surveys (TSS) by year of survey, Amhara, Ethiopia. Favorable TSS are those which returned a trachomatous inflammation-follicular (TF) prevalence of <5% among children ages 1 to 9 years, while recrudescent corresponds to TSS with TF prevalence ≥5%; Figure S2: District-level results from trachoma surveillance surveys (TSS), showing the prevalence of trachomatous inflammation-follicular (TF) compared to prevalence of clean faces in children, Amhara, Ethiopia. Dashed horizontal line corresponds to 5% TF elimination threshold.
