# Supplementary material for: Correlates of Trachoma Recrudescence: Results from 51 District-Level Trachoma Surveillance Surveys in Amhara, Ethiopia
Source: Trop Med Infect Dis. 2024 Dec 5;9(12):298. doi: 10.3390/tropicalmed9120298 (PMC11679309; doi:10.3390/tropicalmed9120298)

Supplemental Figure S1: Histogram of 51 Trachoma Surveillance Surveys (TSS) by year of survey, Amhara, Ethiopia. Favorable TSS are those which returned a trachomatous inflammation-follicular (TF) prevalence of <5% among children ages 1 to 9 years, while recrudescent corresponds to TSS with TF prevalence ≥5%.

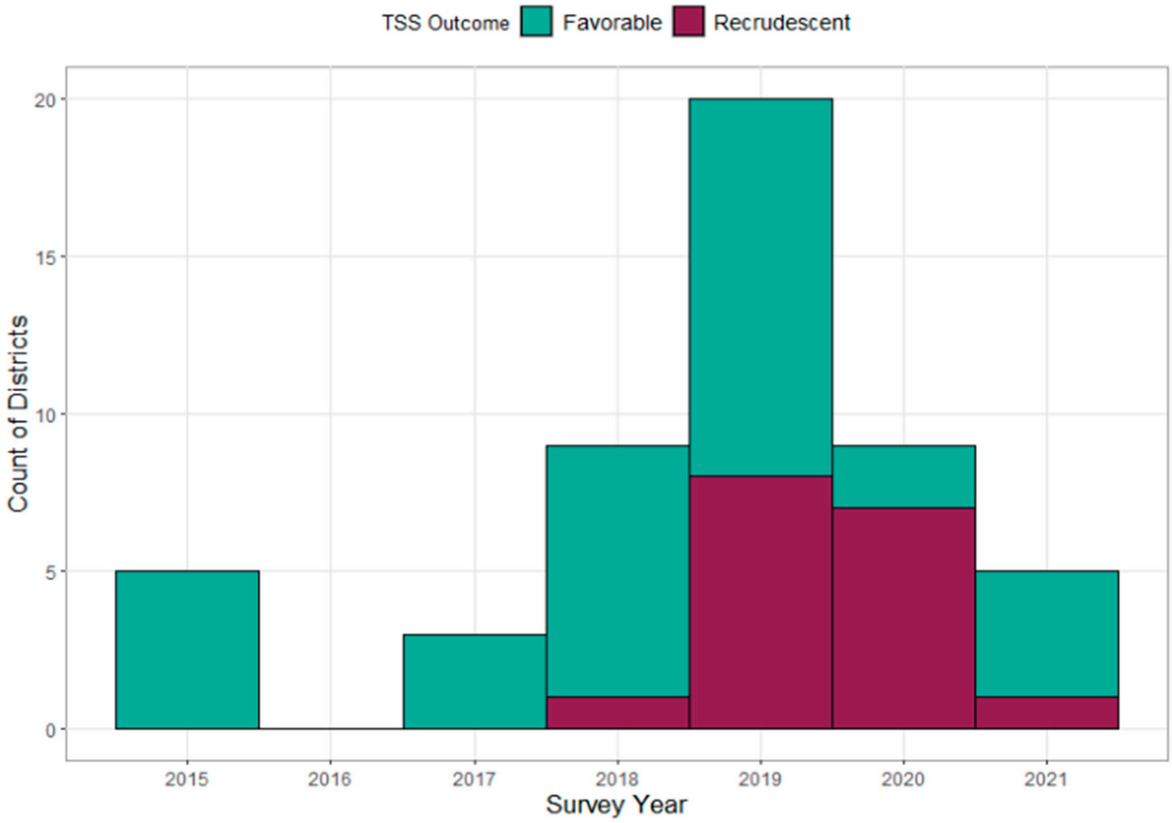

Supplement: Supplementary file 1 [file tropicalmed-09-00298-s001.zip › tropicalmed-3281165-supplementary 1.pdf]
