# Supplementary material for: Correlates of Trachoma Recrudescence: Results from 51 District-Level Trachoma Surveillance Surveys in Amhara, Ethiopia
Source: Trop Med Infect Dis. 2024 Dec 5;9(12):298. doi: 10.3390/tropicalmed9120298 (PMC11679309; doi:10.3390/tropicalmed9120298)

Supplemental Figure S2: District-level results from trachoma surveillance surveys (TSS), showing the prevalence of trachomatous inflammation-follicular (TF) compared to prevalence of clean faces in children, Amhara, Ethiopia. Dashed horizontal line corresponds to 5% TF elimination threshold.

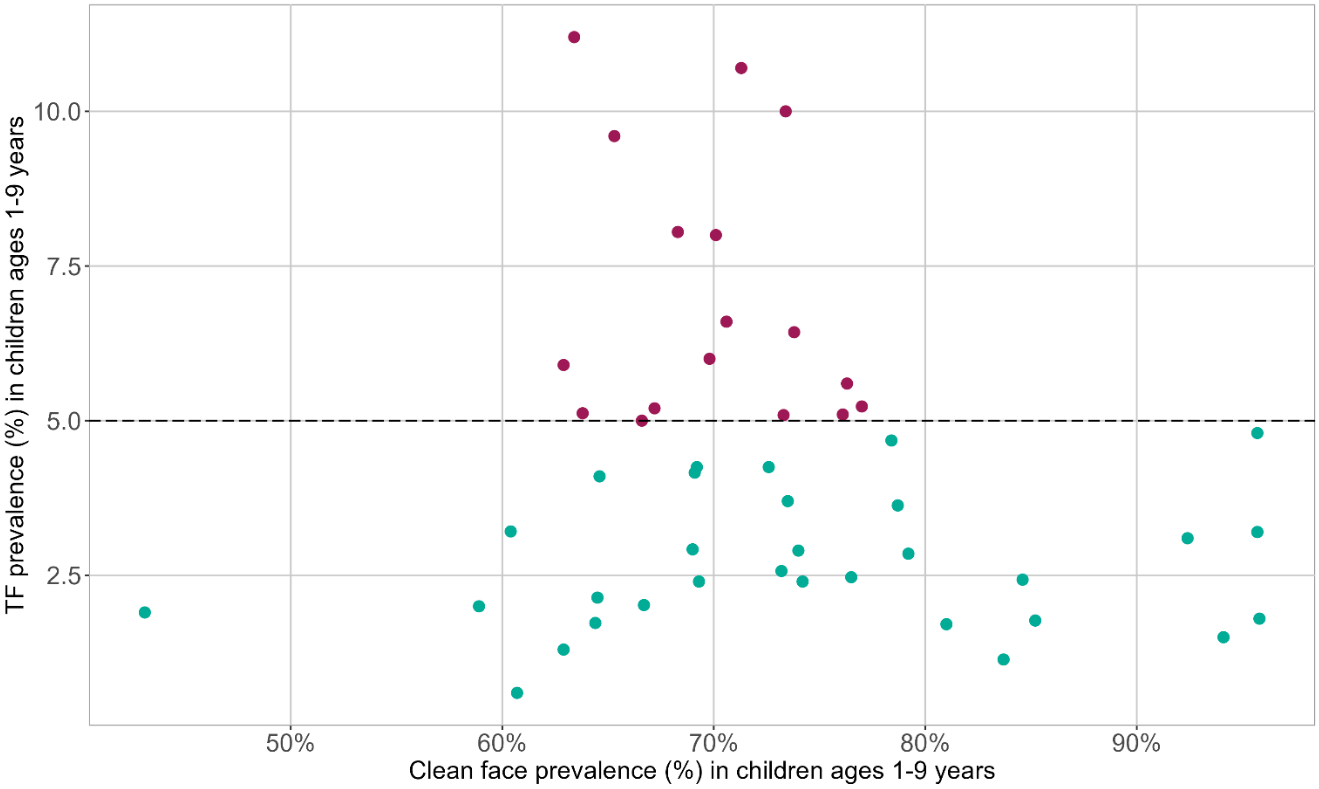

Supplement: Supplementary file 1 [file tropicalmed-09-00298-s001.zip › tropicalmed-3281165-supplementary 2.pdf]
